# In Vivo Selection of a Unique Tandem Repeat Mediated Azole Resistance Mechanism (TR_120_) in *Aspergillus fumigatus cyp51A*, Denmark

**DOI:** 10.3201/eid2503.180297

**Published:** 2019-03

**Authors:** Rasmus K. Hare, Jan B. Gertsen, Karen M.T. Astvad, Kristine B. Degn, Anders Løkke, Marc Stegger, Paal S. Andersen, Lise Kristensen, Maiken C. Arendrup

**Affiliations:** Statens Serum Institut, Copenhagen, Denmark (R.K. Hare, K.M.T. Astvad, M. Stegger, P.S. Andersen, M.C. Arendrup);; Åarhus University Hospital, Åarhus, Denmark (J.B. Gertsen, K.B. Degn, A. Løkke, L. Kristensen);; Rigshospitalet, Copenhagen (M.C. Arendrup);; University of Copenhagen, Copenhagen (M.C. Arendrup)

**Keywords:** promoter TR_120_, azole resistance, whole-genome sequencing, tandem repeat resistance mechanism, in vivo selection, fungal infections, drug resistance, antifungal, Aspergillus fumigatus cyp51A, Denmark, fungi

## Abstract

We report a fatal aspergillosis case in which STR*Af* typing and whole-genome sequencing substantiated in vivo emergence of an azole-resistant *Aspergillus fumigatus* with a 120-bp tandem repeat in the promoter region of *cyp51A*. This event, previously restricted to the environment, challenges current understanding of azole resistance development in *A. fumigatus*.

Azole antifungal drug resistance in *Aspergillus fumigatus* is a concern for patients with aspergillosis because of increased risk for disease and death (*1*). Two routes of acquiring azole resistance have been identified: 1) in vivo, as a consequence of long-term azole treatment; and 2) ex vivo, in the environment, resulting from the use of azole fungicides in crop protection. The underlying mechanisms are primarily linked to structural changes or upregulation of the azole target lanosterol 14 α-demethylase encoded by *cyp51A* (*1*). Most environmentally induced resistance mechanisms involve tandem repeats (TRs) in the promoter region of *cyp51A* coupled with nonsynonymous mutations, TR_34_/L98H and TR_46_/Y121F/T289A (*1*). However, in vivo resistance development has primarily been associated with nonsynonymous mutations in *cyp51A*-inducing amino acid substitutions of hot spots (e.g., G54, G138, M220, and G448) or non–*cyp51A*-mediated mechanisms, but not a tandem repeat (*1*). We describe a clinical case of infection with azole-resistant *A. fumigatus* that acquired a 120-bp tandem repeat (TR_120_) resistance mechanism during long-term azole treatment. The finding was substantiated by whole-genome sequencing (WGS).

## The Study

**Figure 1:**
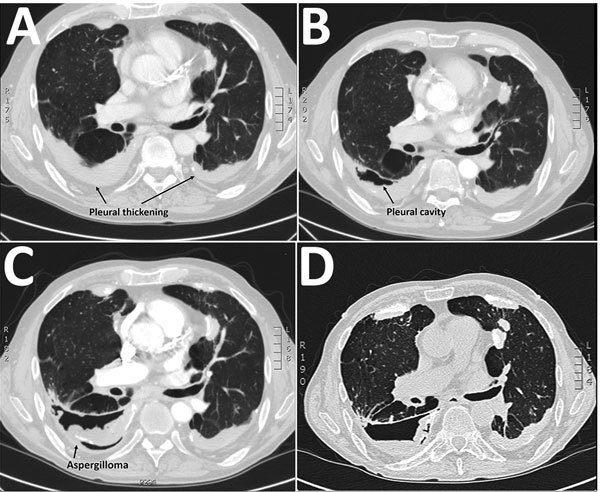
Sequential thoracic computed tomography scan images illustrating the gradual progression from pleural thickening to cavity formation and development of an aspergilloma in a patient with *Aspergillus fumigatus* infection, Denmark, 2013. A) 2011, B) 2012, C) 2014, D) 2016. Scale bar indicates nucleotide substitutions per site.

In 2013, a 69-year-old man who was a former smoker with chronic obstructive pulmonary disease (COPD) and severe airflow obstruction sought care at the University Hospital in Århus, Denmark, because of gradually worsening dyspnea, cough, and expectoration. Previously, in 2011, imaging (Figure 1, panel A) and 2 thoracoscopies had been conducted because of suspicion of malignant mesothelioma. Further histopathologic examination and cultures revealed inflammation but no malignancy or mold infection. Subsequently, in 2012, a fistula between pleura and skin led to a persistent air-containing pleural cavity in the right side (Figure 1, panel B). In 2014, a fungus ball in the pleural cavity was found (Figure 1, panel C). *Aspergillus* IgG titer was 1:25,600 (reference range <1:200), and azole-susceptible *A. fumigatus* was cultured from sputum (P-1, May 2014). Voriconazole (200 mg 2×/d) was given, alternating with posaconazole (300 mg/d) for 2 years until clinical failure, and 2 azole-resistant *A. fumigatus* isolates were cultured from a new sputum sample (P-2 and P-3, June 2016). Despite amphotericin B inhalations followed by liposomal amphotericin B (3 mg/kg 1×/d), the patient died because of severe hemoptysis 1 year later in 2017.

Three *A. fumigatus* patient isolates (P-1, P-2, and P-3) were available for confirmatory species verification, reference susceptibility testing defined by the European Committee on Antimicrobial Susceptibility Testing using protocol for molds (E.Def 9.3), *cyp51A* Sanger sequencing (using wild-type reference sequence AF338659), and genotyping using the short tandem-repeat *Aspergillus fumigatus* (STR*Af*) assay (*2*,*3*) (Table). We included 4 *A. fumigatus* isolates representing relevant *cyp51A* profiles as control strains (SSI-3614 [wild-type], SSI-7828 [TR_34_/L98H], SSI-5717 [TR_46_/Y121F/T289A], and SSI-5197 [F46Y/M172V/E427K]). We detected 3 common Cyp51A variants (F46Y, M172V, and E427K) in the susceptible patient isolate P-1 (GenBank accession no. MG972984). Pan-azole resistance was observed for P-2 and P-3, and both shared *cyp51A* profiles with P-1 but also harbored a TR_120_ mechanism (GenBank accession no. MG972983) in the promoter region (Table). All patient isolates had identical STR*Af* genotypes suggesting that they were isogenic (Table ) (*4*). Furthermore, the STR*Af* profile was unique among *A. fumigatus* isolates genotyped in Denmark (Appendix Figure).

**Table:** *Aspergillus fumigatus* strain characteristics, antimicrobial susceptibility, and molecular data, Denmark, 2013*

Isolate no.	EUCAST-based susceptibility MICs, mg/L			Sanger sequencing: Cyp51A profile§	STR*Af* assay genotyping data:† 2A-2B-2C-3A-3B-3C-4A-4B-4C	WGS data:‡ SNP differences compared with P-1
	VRZ	ITZ	POS			
P-1	1	0.5	0.125	F46Y/M172V/E427K	10–13–10–17–13–8–7–5–6	0
P-2	4	16	0.5	TR_120_/F46Y/M172V/E427K	10–13–10–17–13–8–7–5–6	NA
P-3	4	>16	0.5	TR_120_/F46Y/M172V/E427K	10–13–10–17–13–8–7–5–6	41
SSI-5197	1	1	0.125	F46Y/M172V/E427K	10–15–10–28–13–11–7–5–6	4,968
SSI-7413	0.5	0.25	0.125	WT	21–25–19–28–12–6–20–10–8	105,900
Af293 (13)	1	0.5	0.06	F46Y/M172V/N248T/D255E/E427K	26–18–18–46–21–23–11–10–8	102,727
SSI-5946	4	>16	0.5	TR_34_/L98H	20–21–12–84–10–7–8–9–10	108,901
SSI-5717	>4	0.5	0.25	TR_46_/Y121F/T289A	26–21–16–32–9–10–8–14–10	108,882

*ITZ, itraconazole; NA, not available; POS, posaconazole; SNP, single-nucleotide polymorphism; STR*Af*, short tandem repeat *Aspergillus fumigatus*; VRZ, voriconazole (isavuconazole MICs were equivalent, data not shown); WGS, whole-genome sequencing; WT, wild-type.  †STR*Af* genotyping was performed as previously described (*3*). Underlined STR*Af* markers are shared with P-1. ‡Reference genome coverage ranged from 88.5% to 90.93%. Sequencing depth based on all assembled contigs >1,000 bp ranged from 57.2× to 80.7×; 71.1× for P-1; and 66.3× for P-3.  §TR_34_: GAATCACGCGGTCCGGATGTGTGCTGAGCCGAAT, TR46: **AGTTGTCTA**GAATCACGCGGTCCGGATGTGTGCTGAGCCGAAT**GAA**, TR_120_: TTCTCCTCTAGAAAAAACTCATGAGTGAATAATCGCAGCACCACTCCAG**AGTTGTCTA**GAATCACGCGGTCCGGATGTGTGCTGAGCCGAAT **GAA**AGTTGCCTAATTACTAAGGTGTAGT. GenBank accession numbers are MG972983 with TR_120 _and MG972984 without TR_120_.

**Figure 2:**
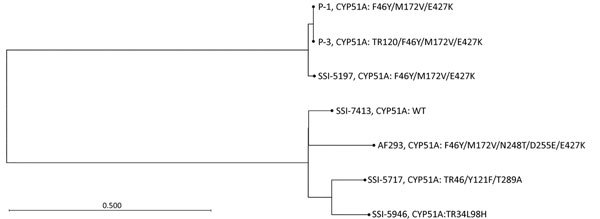
Unrooted phylogenetic tree based on whole-genome sequencing of 2 patient isolates (P-1 and P-3) and 5 reference strains to highlight relatedness between *Aspergillus fumigatus* isolates, Denmark, 2018. We inferred relatedness by using FastTree version 2.1 (*7*) based on a 77.69% core genome. Whole-genome sequencing identified 41 single-nucleotide polymorphism (SNP) differences between P-1 and P-3. We observed subtle differences (<5,000 SNPs) between unrelated patient isolate SSI-5197 and P-1/P-3, whereas >100,000 SNPs differed from P-1/P-3 to the other control strains and Af293. WT, wild-type.

We performed WGS for P-1, P-3, and all control strains to investigate relatedness and other potential mechanisms conferring azole resistance. We subjected total DNA (≈10 ng/µL) to WGS (NextSeq 550; Illumina, https://www.illumina.com) by using Nextera DNA library preparation kit (Illumina) and following the manufacturer’s instructions. We used NASP (*5*) to detect single-nucleotide polymorphisms (SNPs) after removal of duplicated regions in the *A. fumigatus* strain Af293 chromosomes (http://www.aspergillusgenome.org, genome version s03-m05-r09) using NUCmer (*6*). We inferred relatedness by using FastTree version 2.1.5 (*7*) and a 77.69% core genome (Table; Figure 2). To increase resolution, we conducted a subanalysis for P-1 and P-3 (core genome 79.71%), which identified 41 SNP differences; 6 of the SNPs were nonsynonymous in genes with no previous reported association to azole resistance (Appendix Table 1), and 35 were either synonymous or in noncoding regions (Appendix Table 2).

## Conclusions

WGS revealed 41 SNP differences between the susceptible and the resistant patient *A. fumigatus* isolates that evolved during 2 years, similar to a previously described case of in-host microevolution of *A. fumigatus* (*4*). This finding substantiated an isogenic relationship between P-1 and P-3 and demonstrated that the TR_120_ resistance mechanism emerged from P-1, probably during long-term azole therapy. Furthermore, WGS results supported the conclusion that the TR_120_ was the sole mechanism of azole resistance in the azole-resistant patient isolates.

To our knowledge, the TR_120_ is a novel azole-resistance mechanism in *A. fumigatus*, and the in vivo selection of a tandem repeat in the promoter of *cyp51A* is unique. The de novo acquisition of a TR has not previously been shown in vitro or in the environment (i.e., no isolates with L98H or Y121F+T289A combined with wild-type promoters have been reported). However, triplication of an existing TR_34_ on tebuconazole exposure was selected in vitro, and a novel variant, TR_46_^3^, found in clinical and environmental samples, has been derived from sexual mating between TR_46_ parents (*8*,*9*).

Azole resistance involving TRs in the promoter region has been associated exclusively with environmental fungicide selection pressure in *A. fumigatus* and other plant pathogens. Furthermore, although asexual propagation of *A. fumigatus* with TR_34_/L98H or TR_46_/Y121F/T289A resistance mechanisms is widespread in the environment, the extent of de novo selection of TR_34_/L98H and TR_46_/Y121F/T289A is unclear (*10*). One hypothesis describes both environmental resistance mechanisms as being derived from single events of sexual reproduction (in environmental habitats) combining the TR with a *cyp51A* mutant. In addition, sexual reproduction might have led to a high genetic diversity among environmental azole-resistant *A. fumigatus,* which otherwise might have indicated multiple origins (*10*). Our finding might challenge the perception that TR azole-resistance mechanisms are exclusive to the environment and might warrant the question of whether TR_34_/L98H and TR_46_/Y121F/T289A derive from single events.

Hypothetically, the patient might initially have inhaled isogenic isolates with and without TR_120_, the resistant one being undetected. However, a patient being co-infected de novo by a susceptible and an isogenic resistant strain has not been previously reported and is considered highly unlikely.

Long-term and subtherapeutic antifungal treatment might facilitate selection of resistance (*11*). Therapeutic drug monitoring was performed once in this patient but without information if the sample was taken according to guidelines as a trough level (lowest level after dosage). Thus, despite a concentration of 4.3 mg/L (within the recommended trough range), potential subtherapeutic levels during the 200 mg 2×/d dosing scheme cannot be ruled out. The F46Y/M172V/E427K substitutions in Cyp51A, found in both susceptible and resistant isolates, have been suggested to play no role or only a minor role in reduced azole susceptibilities (*12*,*13*). TRs in the promoter region of *cyp51A* have previously been linked to increased *cyp51A* gene expression and MICs because of duplicated *srbA* transcription factor binding motifs (SRE1 and SRE2) leading to increased expression of *cyp51A* (*14*,*15*). Taken together, our data suggest that TR_120_ alone is an important driver of pan-azole resistance at a level comparable to that known to be mediated by the TR_34_/L98H mechanism.

Our WGS results might obviate the desire for in vitro experiments testing the TR_120_ mechanism in laboratory-engineered mutants. Further dissection of the WGS data can help elucidate potential genetic drivers of TR acquisition and add further knowledge as to whether the TR_34_/L98H and TR_46_/Y121F/T289A resistance genotypes derived from a single origin. This report adds another piece to the complex picture of emerging azole-resistant *A. fumigatus* and might serve to stimulate further research.

Appendix Additional information on in vivo selection of a unique tandem repeat mediated azole resistance mechanism (TR_120_) in *Aspergillus fumigatus cyp51A*, Denmark.
