# Supplementary material for: In Vivo Selection of a Unique Tandem Repeat Mediated Azole Resistance Mechanism (TR120) in Aspergillus fumigatus cyp51A, Denmark
Source: Emerg Infect Dis. 2019 Mar;25(3):577–80. doi: 10.3201/eid2503.180297 (PMC6390761; doi:10.3201/eid2503.180297)
Supplement: Appendix — Additional information on in vivo selection of a unique tandem repeat mediated azole resistance mechanism (TR120) in Aspergillus fumigatus cyp51A, Denmark. [file 18-0297-Techapp-s1.pdf]

Article DOI: https://doi.org/10.3201/eid2503.180297

In Vivo Selection of a Unique Tandem Repeat Mediated Azole Resistance Mechanism (TR_{120}) in *Aspergillus fumigatus cyp51A*, Denmark

Appendix

Appendix Table 1. Insight in six non-synonymous SNPs found in the azole resistant isolate P-3 compared to P-1

Gene product	Protein ID AF293	Non-synonymous SNP	Amino acid substitution	Comment
Uncharacterized protein.<br>4-coumarate-CoA ligase	AFUA_1G13110	G1183T	V373*STOP	Precursor of AMP + diphosphate + 4-coumaroyl-CoA–>Coumaroyl-coenzyme A is a central intermediate in the biosynthesis of polyketides such as flavonoids (pigmentation) and other natural organic compounds.<br>Mutation leads to a truncated protein from 566 to 373 aa
Uncharacterized protein.<br>Has domain(s) with predicted nucleus localization	AFUA_2G04780	A1873T	D602G	Ortholog in *C. albicans* is *ENP2*, encodes an essential protein and a heterozygous mutation confers resistance to 5–fluorocytosine (5-FC) and 5-fluorouracil (5-FU). Amino acid position is not conserved but in a conserved region compared to *A. niger* and *A. nidulans* but less for *C. albicans* (12/15 shared amino acids) and the protein sequences have 70%–82% homology.
FLUG	AFUA_3G07140	C650A	Q216*STOP	Upstream activator of conidiation; required for proper regulation of *brlA* transcript levels; calcium induced. White phenotype in *A. nidulans* but not necessarily in *A. fumigatus* (*1*).<br>Mutation leads to a truncated protein from 861 to 216 aa.<br>*A. fumigatus* patient isolate P-3 had a white phenotype.
Uncharacterized protein	AFUA_3G08260	A3192T	K1029M	Orthologs (e.g., *smc1* in *C. albicans*). Has cohesin ATPase activity, double-stranded DNA binding, topological DNA entrapment activity, role in mitotic cohesin loading and condensed nuclear chromosome, centromeric region, nuclear mitotic cohesin complex localization.<br>Amino acid position is in a conserved region compared to other *Aspergilli* (26/31 shared amino acids) and the protein sequences have 84% homology.
Uncharacterized protein.<br>Putative sensor histidine kinase	AFUA_8G06150	G96T	M32I	Ortholog in *A. nidulans* (AN4815).<br>Amino acid position in non-conserved region compared to *A. nidulans* and *A. oryzae* and have 20%–21% protein sequence homology.
Uncharacterized protein.<br>Putative sensor histidine kinase	AFUA_8G06150	C119T	A40V	Same as above.<br>Amino acid position in non-conserved region compared to *A. nidulans* and *A. oryzae* and have 20%–21% protein sequence homology.

*Missense mutation, premature stop-codon leading to a truncated gene product.

Appendix Table 2. Insight in 35 SNPs in non-coding/intergenic regions found in the resistant isolate P-3 compared to P-1. The positions listed are in relation to any annotated gene with the closest proximity, upstream or downstream

SNP position in Af293 (Chromosome::position)	SNP	SNP position in relation to annotated gene (AF293)	Protein function	Comment
Chr1::184219	A → G	571 bp upstream, AFUA_1G00580 (PacG/VIB-1)	Putative DNA binding transcription factor	Intergenic/Promoter region
Chr1::184234	A → G	566 bp upstream, AFUA_1G00580 (PacG/VIB-1)	Same as above	Intergenic/Promoter region
Chr1::38596	G → A	1121 bp upstream, AFUA_1G00200	Uncharacterized	Intergenic
Chr1::38605	A → G	1130 bp upstream, AFUA_1G00200	Uncharacterized	Intergenic
Chr1::51920	G → A	2439 bp downstream, AFUA_1G00220	Uncharacterized	Intergenic
Chr1::964093	T → C	717 bp upstream, AFUA_1G03330	Uncharacterized	Intergenic/Promoter region
Chr1::964119	A → G	691 bp upstream, AFUA_1G03330	Uncharacterized	Intergenic/Promoter region
Chr1::964126	C → T	684 bp upstream, AFUA_1G03330	Uncharacterized	Intergenic/Promoter region
Chr1::964130	C → T	680 bp upstream, AFUA_1G03330	Uncharacterized	Intergenic/Promoter region
Chr1::964137	A → G	673 bp upstream, AFUA_1G03330	Uncharacterized	Intergenic/Promoter region
Chr1::964148	A → G	662 bp upstream, AFUA_1G03330	Uncharacterized	Intergenic/Promoter region
Chr1::964154	A → G	656 bp upstream, AFUA_1G03330	Uncharacterized	Intergenic/Promoter region
Chr1::964159	G → C	651 bp upstream, AFUA_1G03330	Uncharacterized	Intergenic/Promoter region
Chr2::4375395	G → T	344 bp downstream of start codon, AFUA_2G16480	Uncharacterized. Has domain(s) with predicted zinc ion binding activity.	Synonymous SNP
Chr3::1025902	C → T	576 bp downstream, AFUA_3G03740	Uncharacterized. Putative protein kinase	Intergenic/Promoter region
Chr3::1126737	G → A	398 bp downstream, AFUA_3G03950	Uncharacterized (*ste7* in *S. cerevisiae*)	Intergenic/Promoter region. In Af293, this position is an A.
Chr3::1445547	G → A	590 bp downstream startcodon, AFUA_3G05900	MAP kinase kinase	Intron region
Chr3::2483009	G → A	419 bp downstream startcodon, AFUA_3G09710	Uncharacterized (*uga4* in *S. cerevisiae*)	Intron region. In Af293, this position is an A.
Chr3::3962289	C → T	177 bp upstream, AFUA_3G15030	Uncharacterized. Has domains with predicted oxidoreductase, zinc binding dehydrogenase family.	Intergenic/Promoter region
Chr3::686389	A → G	7931 bp upstream, AFUA_3G02670	Uncharacterized (*lys2* in *S. cerevisiae*). Protein similar to nonribosomal peptide synthases (NRPS-like), encoded in a predicted secondary metabolite gene cluster.	Intergenic region
Chr3::686394	T → C	7926 bp upstream, AFUA_3G02670	Same as above.	Intergenic region
Chr4::1902849	T → C	In intron, 702 bp downstream start codon: AFUA_4G07320	Uncharacterized.	Intron region
Chr5::2055153	C → T	Synonymous SNP: 21 bp downstream start codon: AFUA_5G08120	*argJ*, Arginine biosynthesis bifunctional protein.	SrbA-regulated during hypoxia.
Chr5::3843678	A → G	163 bp upstream AFUA_5G14865,	Uncharacterized	Intergenic/Promoter region
Chr5::3843680	T → C	161 bp upstream AFUA_5G14865, conserved hypothetical protein	Uncharacterized	Intergenic/Promoter region
Chr5::3843698	G → T	143 bp upstream AFUA_5G14865, conserved hypothetical protein	Uncharacterized	Intergenic/Promoter region
Chr5::3843704	C → T	137 bp upstream AFUA_5G14865, conserved hypothetical protein	Uncharacterized	Intergenic/Promoter region
Chr5::3856240	A → G	1110 bp upstream, AFUA_5G14920	Uncharacterized	Intergenic region
Chr6::2315039	T → C	735 bp upstream, AFUA_6G09500	Uncharacterized (*caj1* in *S. cerevisiae*).	Intergenic/Promoter region

SNP position in Af293 (Chromosome::position)	SNP	SNP position in relation to annotated gene (AF293)	Protein function	Comment
Chr6::2508784	T → C	828 bp downstream, AFUA_6G10140	Uncharacterized. Has domain(s) with predicted DNA binding, RNA polymerase II transcription factor activity, sequence-specific DNA binding, zinc ion binding activity and role in regulation of transcription, DNA-templated, transcription, DNA-templated.	Intergenic/Promoter region
Chr6::2509191	G → A	1235 bp downstream, AFUA_6G10140	Same as above	Intergenic region
Chr7::1555469	G → A	212 bp downstream, AFUA_7G06350	*phoE*, Putative phosphate transporter, phoB-regulated	In Af293, this position is an A.
Chr7::1644584	C → G	259 bp downstream, AFUA_7G06770 AND 378 bp upstream, AFUA_7G06760	AFUA_7G06770: Conserved protein of unknown function; protein abundantly expressed in conidia; transcript induced in conidia exposed to neutrophils and to human airway epithelial cells. AFUA_7G06760: Uncharacterized.	Intergenic/promoter region
Chr7::2037815	C → T	5870 bp upstream, AFUA_7G08640	Uncharacterized.	Intergenic region
Chr8::1465035	C → T	G111A–>synonymous. Afu8g06150	Uncharacterized. Putative sensor histidine kinase	Synonymous SNP

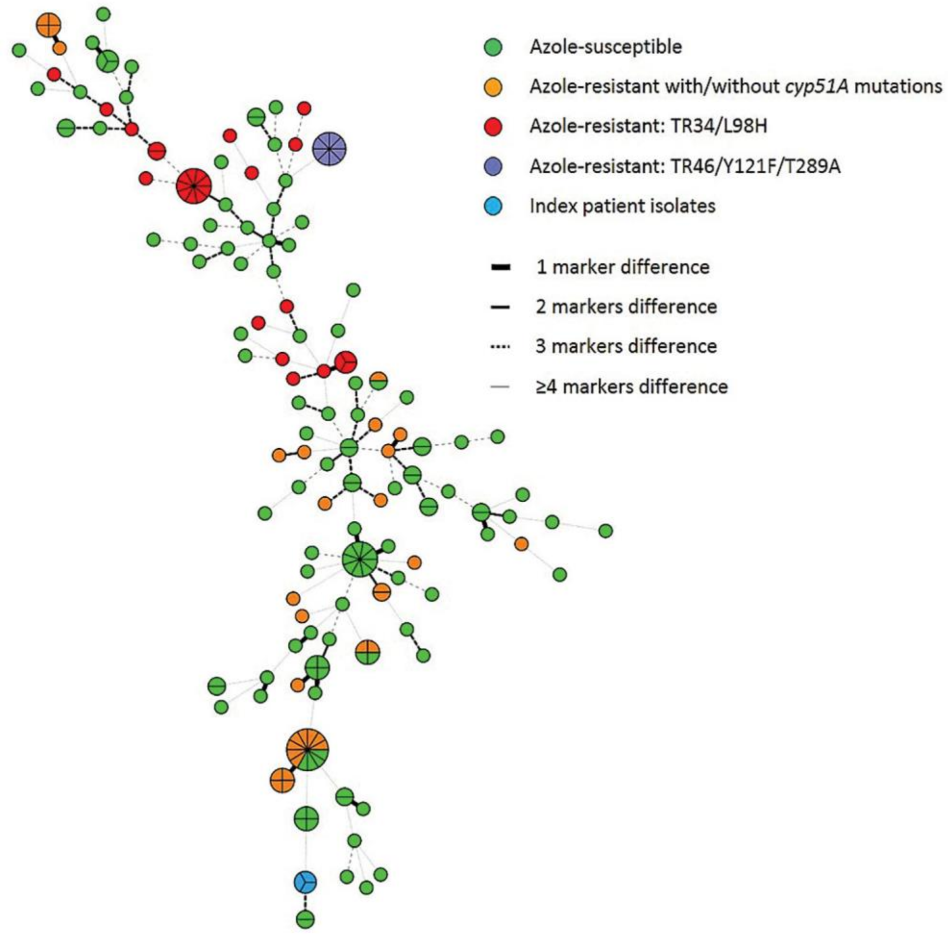

Appendix Figure. Minimum spanning tree created using BioNumerics (v. 7.6, Applied Maths, Sint-Martens-Latem, Belgium) showing Danish STR*Af* genotypes with the three index patient isolates colored in light blue. A total of 122 unique genotypes among 191 clinical and environmental isolates, of which 120 genotypes have previously been described (*2*).

References

1. Mah JH, Yu JH. Upstream and downstream regulation of asexual development in *Aspergillus fumigatus*. Eukaryot Cell. 2006;5:1585–95. PubMed http://dx.doi.org/10.1128/EC.00192-06

2. Jensen RH, Hagen F, Astvad KMT, Tyron A, Meis JF, Arendrup MC. Azole-resistant *Aspergillus fumigatus* in Denmark: a laboratory-based study on resistance mechanisms and genotypes. Clin Microbiol Infect. 2016;22:570.e1–9. PubMed http://dx.doi.org/10.1016/j.cmi.2016.04.001
